# Efficacy of cabozantinib and sunitinib for the treatment of intermediate/poor risk renal cell carcinoma based upon UK real-world data

**DOI:** 10.1016/j.esmorw.2024.100087

**Published:** 2024-10-18

**Authors:** D. Lee, G.J. Melendez-Torres, A. Challapalli, R. Frazer, J. McGrane, A. Bahl

**Affiliations:** 1 PenTAG, University of Exeter, Exeter, UK; 2 Bristol Haematology and Oncology Centre, University Hospitals Bristol and Weston NHS Foundation Trust, Bristol, UK; 3 Velindre Cancer Centre, Cardiff, UK; 4 Royal Cornwall Hospital Oncology Department, Truro, UK

**Keywords:** renal cell carcinoma, cabozantinib, sunitinib, survival, IMDC, UK ROC, real-world, observational

## Abstract

**Background:**

The purpose of this study was to explore the effectiveness of cabozantinib versus sunitinib for the treatment of first-line metastatic renal cell carcinoma in intermediate/poor risk patients.

**Materials and methods:**

Retrospective review of cases between 1 January 2018 and 30 June 2021 across 17 UK centres. Univariable and multivariable Cox proportional hazards modelling to identify prognostic factors. Inverse probability of treatment weighting, to estimate the causal effect of first-line treatment type.

**Results:**

Cabozantinib patients (*n* = 106) had poorer risk status, less prior nephrectomy, shorter time to therapy, and more clear cell histology than sunitinib patients (*n* = 218). More sunitinib patients received a second or third line of subsequent treatment (56% and 23% versus 43% and 13%). Though there was no significant difference between treatments in overall survival (OS) or progression-free survival (PFS) across models, the difference in PFS bordered on significant in a multipredictor analysis (benefit in favour of cabozantinib; *P* = 0.06). When the Kaplan–Meier curves were stratified by risk status (intermediate versus poor), patients had similar OS within the risk groups. PFS appeared to differ with poor risk patients performing better on cabozantinib. Inverse probability of treatment weighting analysis showed little difference from the unadjusted results: OS hazard ratio = 1.119 (95% confidence interval (CI) 0.823-1.521); PFS hazard ratio 0.825 (95% CI 0.636-1.070) for cabozantinib versus sunitinib.

**Conclusions:**

Our results showed no significant difference in either OS or PFS between treatments. Cabozantinib trended towards improved PFS and reduced OS. Decision-making for tyrosine kinase inhibitor monotherapy should consider later-line treatment options. This analysis is of particular relevance as sunitinib is now off-patent meaning that the cost of a course of treatment has considerably reduced.

## Highlights

• No significant PFS/OS difference for sunitinib vs cabozantinib in 1L intermediate/poor risk RCC in large UK real-world cohort.
• Trend towards improved PFS but reduced OS with cabozantinib, likely due to fewer 2nd-line options.
• No prognostic factors identified which significantly impacted on the chance of outcomes differing between the two treatments.

## Introduction

The treatment pathway for first-line advanced renal cell carcinoma (RCC) depends upon the patient’s International Metastatic Renal Cell Carcinoma Database Consortium (IMDC) risk group.^1^^,^^2^ Whilst an increasing number of patients receive immunotherapy-based combinations such as nivolumab plus ipilimumab, axitinib plus avelumab, and pembrolizumab plus lenvatinib a substantial proportion are still treated with tyrosine kinase inhibitor (TKI) monotherapy in UK practice.^3^^,^^4^ A retrospective review of 1,016 intermediate/poor risk patients who started a first-line systemic anticancer treatment between 1 January 2018 and 30 June 2021 found that more than half of the patients were treated with TKI monotherapy.

One of the TKI monotherapies commonly used for intermediate/poor risk patients is cabozantinib; an oral TKI that targets vascular endothelial growth factor receptor (VEGFR), MET, and AXL.^5^ In the first-line setting cabozantinib was approved on the basis of the CABOSUN trial (NCT01835158).^6^

The CABOSUN parallel single-blind trial compared cabozantinib and sunitinib in 157 intermediate/poor risk advanced RCC patients in the USA. CABOSUN found a significant difference in progression-free survival (PFS) in favour of cabozantinib, with hazard ratio (HR) 0.48 [95% confidence interval (CI) 0.31-0.74] on blinded independent committee review and no significant difference in overall survival (OS) with HR 0.80 (95% CI 0.53-1.21). The credibility of the magnitude of PFS benefit observed in CABOSUN was questioned in a recent NICE pilot appraisal for the RCC.^7^ In fact, the magnitude of benefit demonstrated for PFS is numerically greater than that demonstrated by the combination of nivolumab plus cabozantinib in CheckMate 9ER [HR 0.59 (95% CI 0.49-0.71)].^8^ This study was considered to be at high risk of bias^7^ due to issues related to dynamic allocation of treatment, its open-label nature, high attrition rates, and potential conflict from industry funding. It is also noted that the PFS and OS results for sunitinib reported in CABOSUN were lower than in other trials and that the response rate reported in CABOSUN was 20% whereas in subsequent lines response rates of 25%-42% have been observed after immuno-oncology.^9^^,^^10^

Little real-world evidence has been published on the effectiveness of cabozantinib in the first-line setting.^11^ Available data come from a small sample of patients from the IMDC (*n* = 34 only 26 of which were confirmed as intermediate/poor risk)^12^ or focus specifically on non-clear-cell RCC.^13^

We sought to explore the effectiveness of cabozantinib relative to sunitinib for the treatment of first-line RCC in intermediate/poor risk patients using a UK Renal Oncology Collaborative (UKROC) real-world evidence dataset,^14^ using causal inference methods to improve the interpretability and robustness of results.

## Materials and methods

A retrospective review of cases of metastatic RCC (mRCC) was identified across 17 centres in the UK (list of centres in Supplementary Appendix A, available at https://doi.org/10.1016/j.esmorw.2024.100087). The UKROC is a collaboration of UK NHS cancer centres collecting data for real-world evidence in metastatic renal cancer patients.

Patients who started systemic anticancer therapy for mRCC between 1 January 2018 and 30 June 2021 were included and patient characteristics such as gender, IMDC risk group, pattern of metastatic disease at presentation, and lines of therapy were recorded. Patients who were under 18 years of age or who started first-line systematic anticancer therapy (SACT) for mRCC outside the above time period were excluded. As this was a real-world data collection, all histological subtypes and all patterns of initial metastatic sites, including brain metastases, were included. Assessment of progression was based on individual sites analysis of radiographical and clinical data.

Digital records were reviewed by a clinician and data anonymised to ensure that the study was conducted in accordance with the principles of all governance and General Data Protection Regulation (GDPR) regulations. This study was carried out along the ESMO GROW guidelines for real-world data reporting.^15^

Further details relating to the retrospective review are reported elsewhere.^14^^,^^16^

For this analysis the sub-set of patients with intermediate/poor risk status based upon IMDC were included.

### Statistical analysis

Survival data were analysed using Kaplan–Meier curves. Univariable and multivariable Cox proportional hazards modelling was used to estimate the HRs for survival outcomes associated with treatment type and to identify prognostic factors. Models were adjusted for the IMDC risk group, whether or not the cancer was of clear-cell histology, prior nephrectomy status, age at diagnosis, time from diagnosis to first systemic therapy and patterns of metastases. Tests were conduction for interaction between treatment with cabozantinib and each of the potential prognostic variables.

We then used a causal inference method, inverse probability of treatment weighting (IPTW), to better approximate the causal effect of first-line treatment type on disease outcomes. IPTW is one method which can be used to adjust for confounding in observational studies. It uses the propensity score to balance baseline characteristics for the two treatment groups by weighing each individual in the analysis by the inverse probability of receiving their actual treatment exposure. We first constructed stabilised inverse probability weights using a vector of covariates considered based upon clinical expert input to be either prognostic for PFS or OS or moderators of treatment effects: sex, age at first treatment, clear-cell histology, prior nephrectomy, brain metastases and bone metastases, and time from diagnosis to first-line treatment in a logistic regression model for treatment assignment. We then used those weights to re-estimate the Cox proportional hazards model using a corrected sandwich variance estimation method.^17^ All analyses were undertaken in Stata v18.

Progression of disease was defined by clinical teams using clinical and radiological assessment. PFS was calculated from the date of starting first-line SACT to the date of progression. OS was calculated from the first-line SACT to the date of death from any cause or, for surviving patients, to the date of last follow-up.

## Results

Of the patients with intermediate/poor risk; 106 received cabozantinib at first line and 218 received sunitinib (Table 1). Patients receiving cabozantinib were more likely to have poor, rather than intermediate, IDMC risk status (50% versus 28%), were less likely to have had a prior nephrectomy (36% versus 50%), and had a shorter time from diagnosis to first systemic therapy (66 days shorter) all of which are predictive of poorer outcomes. When we looked at the time from diagnosis to first systemic therapy based on prior nephrectomy status, the mean time for patients who did have a nephrectomy was 46 days for patients receiving cabozantinib who did not have a nephrectomy and 277 days for those who did compared with 91 days for patients receiving sunitinib who did not have a nephrectomy and 303 days for those who did, which indicates that patients receiving cabozantinib may have been considered to have more aggressive disease. Patients receiving cabozantinib were, however, more likely to be of clear-cell histology (86% versus 77%) which is predictive of better outcomes. Patients receiving both treatments had similar profiles in terms of age, gender, and the location of metastases. Data were mature for both treatments with >80% of patients having experienced a progression event and >60% having died. More patients in the cabozantinib arm had progression recorded as the primary reason for discontinuation than in the sunitinib arm (74% versus 60%), and the proportion discontinuing due to toxicity was slightly higher for sunitinib (17% versus 24%).

**Table 1:** Patient and tumour characteristics

	Cabozantinib *n* = 106	Sunitinib *n* = 218	Difference in baseline characteristics. Cabozantinib versus sunitinib
Deaths observed, *n* (%)	67 (63.2)	155 (71.1)	—
PFS events observed, *n* (%)	87 (82.1)	200 (91.7)	—
Age at diagnosis of metastatic disease, mean years (SE), *n* = 319	63.6 (0.926)	64.2 (0.757)	−0.6
Proportion female, *n* (%)	30 (28.3)	69 (31.7)	−3.3%
IMDC risk category			
Intermediate, *n* (%)	53 (50)	156 (71.6)	−21.6%
Poor, *n* (%)	53 (50)	62 (28.4)	+21.6%
Clear cell histology, *n* (%)	91 (85.8)	167 (76.6)	+9.2%
Prior nephrectomy, *n* (%)	38 (35.8)	109 (50)	−14.2%
Time from diagnosis to first systemic therapy, days (SE)	129 (25.9)	195 (30.4)	−66
Bone metastases, *n* (%)	39 (36.8)	67 (30.7)	+6.1%
Brain metastases, *n* (%)	11 (10.4)	17 (7.8)	+2.6%
Sarcomatoid changes, *n* (%)	8/99 (8.1)	10/196 (5.1)	+3.0%
Primary reason for discontinuation			
Death	5 (4.2)	8 (5.9)	−1.7%
Progressive disease	51 (74.2)	141 (60)	+14.2%
Toxicity	20 (17.4)	33 (23.5)	−6.2%
Patient choice	3 (1.1)	2 (3.5)	−2.5%
Other	5 (2.1)	4 (5.9)	−3.8%
Not recorded	1 (1.1)	2 (1.2)	−0.1%
Mean lines of treatment received	1.6	2.2	—
Second line treatment, *n* (%)	46 (43.3)	121 (55.5)	—
Axitinib, *n*	1	12	
Cabozantinib, *n*	1	42	
Lenvatinib + everolimus, *n*	3	1	
Nivolumab + ipilimumab, *n*	9	0	
Nivolumab, *n*	31	63	
Pazopanib, *n*	0	1	
Sunitinib, *n*	1	0	
Tivozanib, *n*	0	2	
Third-line treatment, *n* (%)	14 (13.2)	51 (23.4)	—
Axitinib, *n*	4	5	
Cabozantinib, *n*	0	29	
Everolimus, *n*	0	1	
Lenvatinib + everolimus, *n*	5	0	
Nivolumab, *n*	1	16	
Sunitinib, *n*	4	0	
Fourth-line treatment *n* (%)	6 (5.7)	10 (4.6)	—
Axitinib, *n*	3	5	
Cabozantinib, *n*	0	1	
Everolimus, *n*	2	4	
Other, *n*	1	0	

IMDC, International Metastatic Renal Cell Carcinoma Database Consortium; PFS, progression-free survival; SE, standard error.

**Figure 1:**
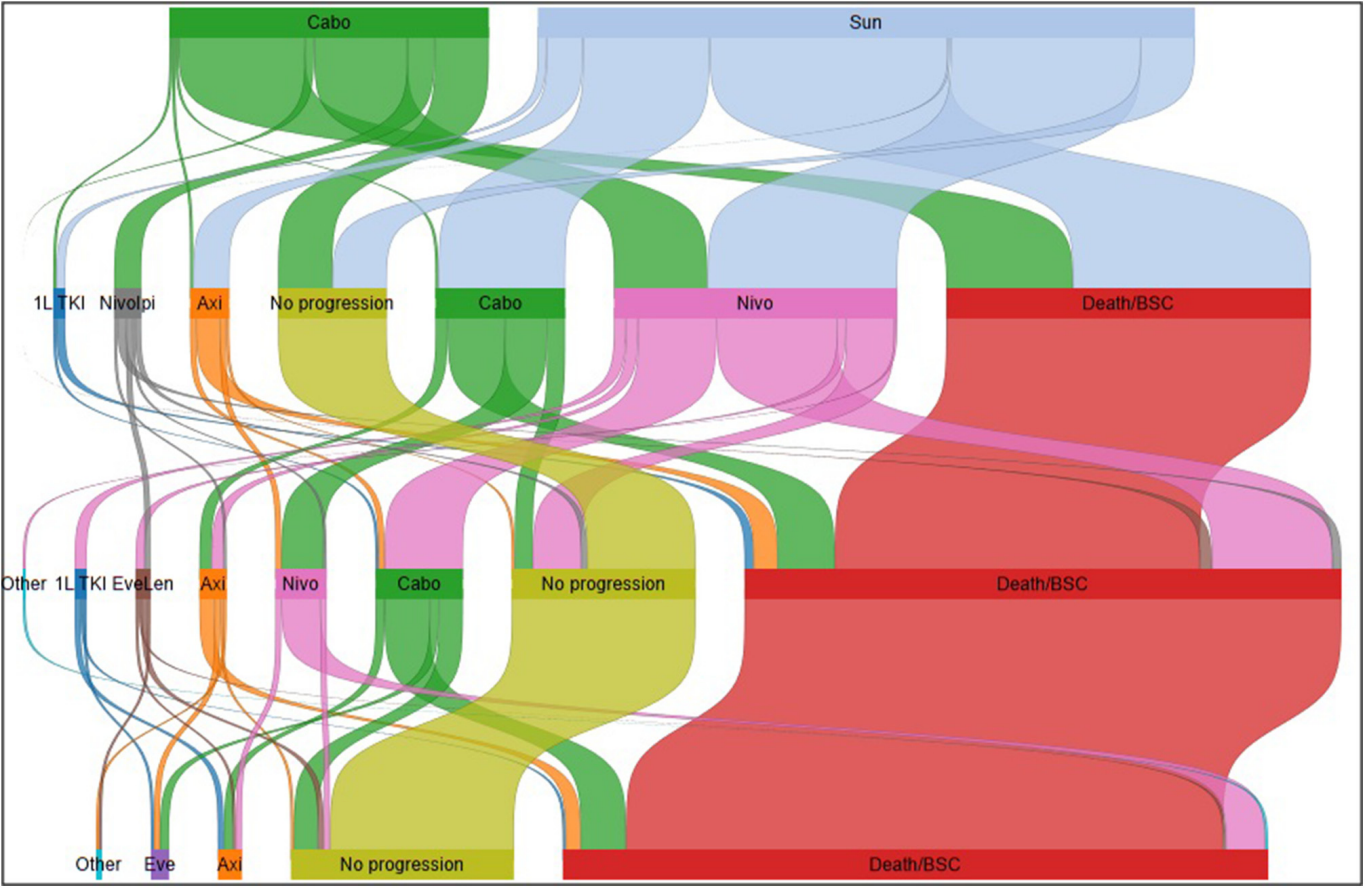
**Sankey plot of treatments received**. 1L TKI, first-line tyrosine kinase inhibitor (sunitinib, pazopanib, tivozanib); Axi, axitinib; BSC, best supportive care; Cabo, cabozantinib Eve, everolimus; Eve/Len; everolimus + lenvatinib; Nivo, nivolumab; NivoIpi, nivolumab + ipilimumab; Sun, sunitinib.

More patients in the sunitinib arm received a second or third line of subsequent treatment than in the cabozantinib arm (56% and 23% versus 43% and 13%). The mean number of lines received was 1.6 for cabozantinib compared with 2.2 for sunitinib. The types of subsequent treatment received were generally similar (Figure 1, Table 1) except that cabozantinib was frequently used after sunitinib and nine patients received nivolumab plus ipilimumab as a second-line treatment after cabozantinib. The use of nivolumab plus ipilimumab off-label as a second-line treatment is due to a COVID NICE guidance allowance which was an exception to standard use and had no clear impact on survival for patients who received at least two lines of treatment (Supplementary Figure S7, available at https://doi.org/10.1016/j.esmorw.2024.100087). Only 52% of patients received a checkpoint inhibitor after sunitinib compared with 89% after cabozantinib. Nivolumab has been available for previously treated patients across England since November 2016, following NICE recommendation.^18^ The UKROC dataset reflects the national picture^3^^,^^4^ in that treatment patterns are highly variable across England, in this case potentially due to a belief by some within the clinical community that recurrent TKI use is preferrable to use of checkpoint inhibitors, even following publication of the CheckMate 025 trial (NCT01668784).^19^ There did not appear to be any correlation between the year of metastatic diagnosis and whether or not a programmed cell death protein 1 (PD-1) inhibitor was used at second line (Supplementary Table S1, available at https://doi.org/10.1016/j.esmorw.2024.100087). Nivolumab has never been compared head-to-head with cabozantinib for use after a first-line TKI.

**Figure 2:**
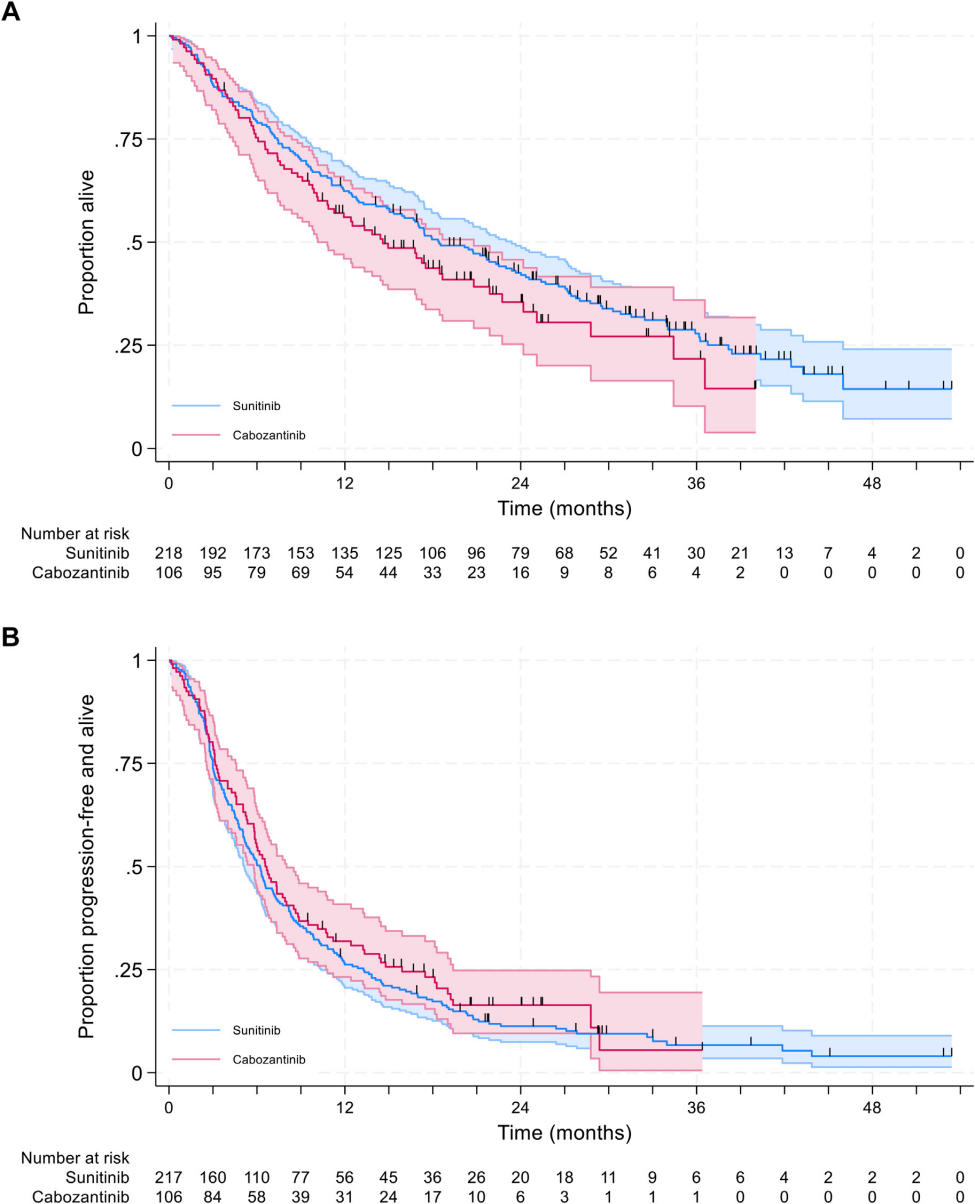
**Unadjusted overall survival by treatment.** (A) Overall survival. (B) Progression-free survival. Note one patient on the sunitinib arm had their PFS event time recorded as day 0 and was therefore excluded from the analysis. PFS, Progression-free survival.

Patients who received cabozantinib had numerically lower OS but numerically higher PFS than patients who received sunitinib in adjusted analysis although confidence intervals overlapped for the observed time horizon (Figure 2). Median OS was 18.4 months for sunitinib and 14.6 months for cabozantinib with 62.4% versus 56.0%; 42.6% versus 35.5%, and 27.8% versus 21.7% surviving to 12, 24, and 36 months for sunitinib versus cabozantinib. Median PFS was 6.2 months for sunitinib and 6.6 months for cabozantinib with 26.2% versus 31.9%; 11.3% versus 16.4%, and 6.7% versus 5.5% remaining alive and progression free at 12, 24, and 36 months for sunitinib versus cabozantinib.

No significant difference was observed between treatments in the unadjusted Cox proportional hazards analysis for either endpoint in either univariate or multivariate analysis (Tables 2 and 3) although the difference in PFS bordered on significant in the multivariate analysis (benefit in favour of cabozantinib; *P* = 0.06).

**Table 2:** Multivariate cox proportional hazards analysis

	OS		PFS	
	Hazard ratio (95% CI)	*P* value	Hazard ratio (95% CI)	*P* value
Treatment with cabozantinib	1.077 (0.792-1.466)	0.636	0.772 (0.590-1.011)	0.060
IMDC score: poor risk	**1.996 (1.489**-**2.675)**	**<0.001**	**1.911 (1.467**-**2.489)**	**<0.001**
Prior nephrectomy: yes	0.810 (0.602-1.090)	0.164	0.925 (0.714-1.198)	0.554
Male	0.980 (0.733-1.311)	0.894	0.938 (0.726-1.213)	0.626
Age at start of first-line systemic treatment, years	1.007 (0.994-1.021)	0.263	0.999 (0.988-1.010)	0.855
Time between diagnosis and first-line systemic treatment, days	**1.000 (1.000**-**1.001)**	**0.035**	1.000 (1.000-1.000)	0.819
Clear-cell histology	**0.659 (0.476**-**0.914)**	**0.012**	**0.739 (0.552**-**0.991)**	**0.043**
Bone metastases	1.269 (0.953-1.690)	0.103	0.936 (0.725-1.209)	0.613
Brain metastases	1.254 (0.771-2.039)	0.362	1.000 (0.646-1.549)	1.000

CI, confidence interval; IMDC, International Metastatic Renal Cell Carcinoma Database Consortium; OS, overall survival; PFS, progression-free survival.

Note: significant predictors at the *P* < 0.05 level highlighted in bold.

**Table 3:** Comparison of IPTW and unadjusted Cox proportional hazards analysis

	Hazard ratio cabozantinib versus sunitinib (95% CI)	*P* value
Unadjusted		
OS	1.219 (0.910-1.632)	0.183
PFS	0.891 (0.692-1.148)	0.373
IPTW adjusted (average treatment effect)		
OS	1.119 (0.823-1.521)	0.474
PFS	0.825 (0.636-1.070)	0.146

CI, confidence interval; IPTW, inverse probability treatment weighting; OS, overall survival; PFS, progression-free survival.

IMDC risk score and clear-cell histology were both highly prognostic for OS and PFS; the time between diagnosis and initiation of systemic treatment was also prognostic for OS (but not PFS). None of the other potential predictors were found to have a significant prognostic impact on outcomes.

When the Kaplan–Meier curves were stratified by IMDC risk status (intermediate versus poor) patients had similar OS across the two treatments within the risk groups (Supplementary Figure S5, available at https://doi.org/10.1016/j.esmorw.2024.100087); PFS, however, appeared to differ with poor risk patients performing better on cabozantinib. Sample sizes for PFS in the poor risk subgroup were, however, small; particularly for later time points in the analysis. When the Kaplan–Meier curves were stratified by histology (Supplementary Figure S6, available at https://doi.org/10.1016/j.esmorw.2024.100087) patients had similar PFS across the two treatments by histology, however, OS appeared to differ with poorer outcomes for cabozantinib for both histologies, although sample sizes were small for non-clear cell RCC.

No significant interactions between treatment with cabozantinib and any of the potential prognostic variables and outcomes were found (Supplementary Table S2, available at https://doi.org/10.1016/j.esmorw.2024.100087). The closest to significance was prior nephrectomy status for PFS (HR 0.61 for the interaction term, *P* = 0.075); no other tests had *P* < 0.1. In a multivariable model adjusting for the interaction between cabozantinib and prior nephrectomy status (Supplementary Table S3, available at https://doi.org/10.1016/j.esmorw.2024.100087), a significant benefit to treatment with cabozantinib was found (HR = 0.63, *P* = 0.011), however, the model did not provide an improved goodness of fit (Bayesian information criterion 2879.4 versus 2876.7). Given conflicting literature on whether cabozantinib has particular benefit for patients with bone metastases we paid particular attention to whether there was a significant interaction between treatment with cabozantinib and the presence of bone metastases.^20^, ^21^, ^22^ We did not find any evidence of this when adding the interaction as an additional term to the multivariable model (HR 1.10, *P* = 0.74 for OS, HR = 1.06, *P* = 0.83 for PFS).

The results of the Schoenfeld residual test did not rule out proportional hazards as a reasonable assumption (*P* = 0.952 for OS, *P* = 0.884 for PFS) and a log-log plot did not provide evidence of violation of proportional hazards which allows confidence in using the Cox regression results (Supplementary Figure S1, available at https://doi.org/10.1016/j.esmorw.2024.100087).

The IPTW analysis showed little difference from the unadjusted results (Table 2). For both OS and PFS, HRs moved slightly more in favour of cabozantinib as would be expected given the generally poorer prognosis of patients at baseline, however, OS remains in favour of sunitinib [HR = 1.119 (95% CI 0.823-1.521)] with PFS in favour of cabozantinib [HR = 0.825 (95% CI 0.636-1.070)]. There was no evidence of violation of the overlap assumption (Supplementary Figure S2, available at https://doi.org/10.1016/j.esmorw.2024.100087) and the specification test (Hansen’s J-statistic^23^) did not indicate that the null hypothesis that the propensity score model is correctly specified should be rejected (*P* = 0.8775 for OS, *P* = 0.8808 for PFS). The weighting achieved a good balance between covariates (Supplementary Tables S4 and S5, available at https://doi.org/10.1016/j.esmorw.2024.100087) and no weights of >3 were observed (Supplementary Figure S3 shows the Kaplan Meier curves following weighting and Supplementary Figure S4 shows the fitted weight density, available at https://doi.org/10.1016/j.esmorw.2024.100087). In particular, very low standardised differences (<0.01) are reported for IMDC risk status and histology which are the covariates with the greatest prognostic impact.

## Discussion

We found no significant difference in outcomes between cabozantinib and sunitinib for either PFS or OS in our real-world dataset in either adjusted or unadjusted analyses. We found a trend towards improved PFS for cabozantinib and reduced OS. Better performance on PFS than OS was also found in the CABOSUN RCT which found a significant difference in PFS in favour of cabozantinib and no significant difference in OS. The point estimate for OS in CABOSUN favoured cabozantinib, however, whereas the point estimate in our analysis favours sunitinib.

Within our dataset a greater proportion of patients received second- and third-line treatment after sunitinib than after cabozantinib (56% versus 43% received a second-line treatment) with the majority of those who got a second-line treatment receiving PD-1 checkpoint inhibitors after both treatments (52% versus 89%) and cabozantinib also being a frequently used treatment after sunitinib (34%). Within the CABOSUN RCT, receipt of subsequent treatment was more balanced between the arms with 65% and 64% receiving any subsequent treatment after cabozantinib and sunitinib, respectively. The majority of subsequent treatment received in CABOSUN was TKI monotherapy as opposed to PD-1 checkpoint inhibitors (only 18% and 19%).^6^ The imbalance in subsequent treatments received may impact on outcomes, but also reflects current practice given the availability of nivolumab since November 2016.

Compared with our dataset, patients in CABOSUN were less likely to be in the IMDC poor risk group (19% versus 35%), to have had a prior nephrectomy (27% versus 45%), and to have non-clear-cell histology (0% versus 20%) indicating a generally better prognosis in the RCT as would be expected given the studies inclusion criteria. Data were not presented in CABOSUN on the time to first systemic treatment.

When we looked at the data by risk subgroup OS was similar for the two treatments within each risk group. We did not find any evidence that histology, prior nephrectomy or the presence of bone metastases had any impact on the relative benefits of the two treatments.

For the cohort of patients where single-agent TKI is appropriate due to comorbidity, contraindications to immunotherapy and/or patient preference these data do not indicate any strong reason to prefer one treatment over another on the basis of clinical effectiveness alone.

This analysis is of particular relevance as sunitinib is now off-patent meaning that the cost of a course of treatment has considerably reduced. Based upon prices in the electronic Marking Information Tool (eMIT), between July 2022 and June 2023 the cost was £812.32 for a pack of 28 × 50 mg tablets; in the previous 6 months of data in eMIT the cost was £1388.77.^24^ This compares with a list price of £5,143 for a pack of 30 tablets of cabozantinib and a cost per 28 days of £9,696 for pembrolizumab with lenvatinib and £10,066 for nivolumab with cabozantinib, although confidential discounts do apply to all of these treatments.^25^

The strength of this analysis is the extensive modelling of confounding factors to give confidence in the robustness of the results. This is a large cohort, with good quality data and minimal missing values in the immunotherapy era with a large spread of NHS centres across the UK giving a good representation of the UK treatment landscape. Our study evaluated the use of cabozantinib in the first-line setting. Whilst a number of studies such as CABOREAL are available looking at outcomes for patients treated at later lines, evidence in the first-line setting so far has been very limited.^9^, ^10^, ^11^, ^12^, ^13^^,^^26^ The only study which included more than 10 patients in the first-line setting and provided outcomes specific to the intermediate / poor risk setting identified for comparison was a multicentre, retrospective, cohort study in non-clear-cell RCC in the USA which included 22 first-line patients and found a median time to treatment failure of 7.6 months (95% CI 5.5-17.2 months); similar to the 6.6 month PFS reported here.^13^

This large UK dataset has several limitations which are inherent to the real-world aspect of data collection and analysis. This was a retrospective data collection. There were no data collected for response rate and limited data collected regarding treatment toxicity and patient comorbidity and their impact on treatment choice. There were a limited number of unmeasured potential confounders not collected in the dataset which were identified in key publications^27^, ^28^, ^29^, ^30^, ^31^, ^32^, ^33^, ^34^ on prognostic factors in advanced RCC: performance status and laboratory parameters such as such as haemoglobin levels, lactate dehydrogenase levels, and calcium levels. The omission of these as independent variables within the dataset was not considered likely to have a major impact on results as they are captured within the IMDC risk score. Due to the evolving nature of this treatment space we now also have additional first-line combinations which were not in routine use during the time frame of this study and also adjuvant pembrolizumab in the non-metastatic setting^35^ which may impact treatment choices.

## Conclusion

Our results showed no significant difference in either OS or PFS between treatments. In line with the CABOSUN trial, our results indicate a trend towards improved PFS for cabozantinib relative to sunitinib. Our results, however, indicate a trend towards reduced, rather than improved, OS. This may be a result of reduced treatment options after cabozantinib, as a large number of patients receiving sunitinib at first line went on to receive cabozantinib at second line. When deciding which TKI monotherapy to give in first line for patients who are not prescribed immuno-oncology combination therapy the impact on the availability of later line treatment options should be considered.
