# Supplementary material for: Efficacy of cabozantinib and sunitinib for the treatment of intermediate/poor risk renal cell carcinoma based upon UK real-world data
Source: ESMO Real World Data Digit Oncol. 2024 Oct 18;6:100087. doi: 10.1016/j.esmorw.2024.100087 (PMC12836660; doi:10.1016/j.esmorw.2024.100087)
Supplement: Supplementary data [file mmc2.docx]

# Supplementary Appendix A

**Participating UKROC Centres**

1. Bristol Oncology Centre, Bristol Royal Infirmary – Amar Challapalli, Amit Bahl
2. Velindre Cancer Centre, Cardiff – Ricky Frazer
3. Sunrise Oncology Centre, Royal Cornwall Hospital – John McGrane
4. Edinburgh Oncology Centre, Western General Hospital – Dhruv Abhi
5. Northern Ireland Cancer Centre, Belfast City Hospital – Alison Clayton [Alison.clayton@belfasttrust.hscni.net](mailto:Alison.clayton@belfasttrust.hscni.net)
6. Newcastle Cancer Centre - JIANG, Xue (THE NEWCASTLE UPON TYNE HOSPITALS NHS FOUNDATION TRUST) <xue.jiang@nhs.net>
7. Mount Vernon Cancer Centre, Middlesex – Anand Sharma
8. University Hospital of Southampton – [Eleanor.Jones2@uhs.nhs.uk](mailto:Eleanor.Jones2@uhs.nhs.uk) – Eleanor Jones
9. Oxford University Hospital – Mark Tuthill
10. Lancashire Teaching Hospital, Preston - Alexandra ferrera
11. Musgrove Park Hospital, Taunton – Gihan Ratnayake
12. Torbay & South Devon Foundation Hospital – Claire Dyke, [Claire.dyke@nhs.net](mailto:Claire.dyke@nhs.net) (oncology SpR)
13. Royal Devon & Exeter Hospital – Vicky Ford
14. University Hospital Plymouth – Dominique Parslow
15. South West Wales Cancer Centre, Swansea – Sing-Yu Moorcroft
16. Hull Hospital – Justin Liu
17. Pool Hospital – Tom Geldart

# Supplementary Appendix B

**Figure B.1 Log-log plot**

1. Overall survival

1. Progression-free survival

**Figure B.2 Overlap: estimated densities of the probability of getting each treatment level**

1. Overall survival

1. Progression-free survival

**Figure B.3 Kaplan Meier plot following weighting**

1. Overall survival

1. Progression-free survival

**Figure B.4 Fitted weight density**

1. Overall survival

1. Progression-free survival

**Figure B.5 Kaplan Meier Curves Stratified by Risk Subgroup**

1. Overall survival

1. Progression-free survival

**Figure B.6 Kaplan Meier Curves Stratified by Histology**

1. Overall survival

1. Progression-free survival

**Figure B.7 Impact of Subsequent Nivolumab plus Ipilimumab on Survival**

**Table B.1 Type of subsequent treatment received by year of diagnosis**

|  | **Cabozantinib** | | **Sunitinib** | |
| --- | --- | --- | --- | --- |
| **Year of metastatic diagnosis** | **PD-1 inhibitor** | **Other** | **PD-1 inhibitor** | **Other** |
| 2009 | 0 | 0 | 0 | 1 |
| 2013 | 0 | 0 | 0 | 1 |
| 2016 | 0 | 1 | 0 | 0 |
| 2017 | 1 | 0 | 10 | 7 |
| 2018 | 9 | 0 | 31 | 27 |
| 2019 | 6 | 2 | 11 | 17 |
| 2020 | 18 | 1 | 11 | 5 |
| 2021 | 6 | 2 | 0 | 0 |

**Table B.2 Results of treatment interaction tests (p value)**

|  | **PFS** | **OS** |
| --- | --- | --- |
| IMDC score: poor risk | 0.415 | 0.443 |
| Prior nephrectomy: yes | 0.075 | 0.358 |
| Male | 0.876 | 0.915 |
| Age at start of 1st line systemic treatment | 0.901 | 0.741 |
| Time between diagnosis and 1st line systemic treatment | 0.612 | 0.467 |
| Clear-cell histology | 0.414 | 0.126 |
| Bone metastases | 0.833 | 0.744 |
| Brain metastases | 0.303 | 0.246 |

Abbreviations: IMDC, International Metastatic RCC Database Consortium

**Table B.3 Multivariate cox proportional hazards analysis for PFS (sensitivity analysis including treatment interaction effect with prior nephrectomy)**

|  | **Hazard ratio (95% CI)** | **P value** |
| --- | --- | --- |
| **Treatment with cabozantinib** | **0.634 (0.446, 0.899)** | **0.011** |
| **IMDC score: poor risk** | **2.006 (1.534, 2.623)** | **<0.001** |
| Prior nephrectomy: yes | 1.321 (0.831, 2.100) | 0.239 |
| Male | 0.935 (0.723, 1.208) | 0.606 |
| Age at start of 1st line systemic treatment, years | 0.998 (0.986, 1.009) | 0.695 |
| Time between diagnosis and 1st line systemic treatment, days | 1.000 (1.000, 1.000) | 0.792 |
| Clear-cell histology | 0.758 (0.565, 1.018) | 0.065 |
| Bone metastases | 0.946 (0.733, 1.222) | 0.673 |
| Brain metastases | 0.979 (0.631, 1.519) | 0.924 |
| Treatment interaction effect: prior nephrectomy and treatment with cabozantinib | 0.613 (0.357, 1.051) | 0.075 |

Abbreviations: IMDC, International Metastatic RCC Database Consortium

**Note: significant predictors at the p<0.05 level highlighted in bold**

**Table B.4 Covariate balance, overall survival**

|  | **Standardised differences** | | **Variance ratio** | |
| --- | --- | --- | --- | --- |
|  | **Raw** | **Weighted** | **Raw** | **Weighted** |
| IMDC score: poor risk | 0.451103 | -0.00739 | 1.234402 | 0.995326 |
| Prior nephrectomy: yes | -0.28786 | 0.046335 | 0.924401 | 1.005348 |
| Male | 0.072903 | 0.002915 | 0.942585 | 0.997491 |
| Age at start of 1st line systemic treatment | -0.06723 | -0.01694 | 0.722748 | 0.696958 |
| Time between diagnosis and 1st line systemic treatment | -0.17733 | -0.01356 | 0.353023 | 0.642721 |
| Clear-cell histology | 0.237608 | -0.00864 | 0.681188 | 1.012623 |
| Bone metastases | 0.127919 | -0.0138 | 1.097763 | 0.989291 |
| Brain metastases | 0.089492 | 0.011434 | 1.299847 | 1.032947 |

Abbreviations: IMDC, International Metastatic RCC Database Consortium

**Table B.5 Covariate balance, progression-free survival**

|  | **Standardised differences** | | **Variance ratio** | |
| --- | --- | --- | --- | --- |
|  | **Raw** | **Weighted** | **Raw** | **Weighted** |
| IMDC score: poor risk | 0.448081 | -0.00711 | 1.230968 | 0.995534 |
| Prior nephrectomy: yes | -0.28317 | 0.046312 | 0.924401 | 1.005649 |
| Male | 0.076029 | 0.003371 | 0.940248 | 0.997123 |
| Age at start of 1st line systemic treatment | -0.06506 | -0.01618 | 0.720012 | 0.69587 |
| Time between diagnosis and 1st line systemic treatment | -0.1755 | -0.01424 | 0.351521 | 0.63631 |
| Clear-cell histology | 0.240149 | -0.00775 | 0.679005 | 1.011286 |
| Bone metastases | 0.124852 | -0.01399 | 1.094943 | 0.989208 |
| Brain metastases | 0.088164 | 0.010716 | 1.294362 | 1.030818 |

Abbreviations: IMDC, International Metastatic RCC Database Consortium
